# Timing of Urinary Catheter Removal Following Kidney Transplantation: A Retrospective Study

**DOI:** 10.1016/j.xkme.2022.100484

**Published:** 2022-05-19

**Authors:** Itai Bezherano, Liise K. Kayler

**Affiliations:** 1 Jacobs School of Medicine and Biomedical Sciences at the University at Buffalo, Buffalo, New York; 2 Transplant and Kidney Care Regional Center of Excellence, Erie County Medical Center, Buffalo, New York; 3 Department of Surgery, University at Buffalo, Buffalo, New York

To the Editor:

Kidney transplant outcomes have improved over recent decades because of medical and surgical advances.^1^ Bladder catheterization has remained constant.^2^, ^3^, ^4^, ^5^, ^6^ Foley catheter use facilitates bladder identification by retrograde saline infusion into the bladder before anastomosis. After transplantation, the Foley catheter prevents tension on the new anastomosis by continuous drainage of urine and is typically maintained in place for 4-10 days to prevent urinary complications.^2^^,^^5^ However, the optimal Foley duration to minimize urinary complications is unknown.

Earlier Foley catheter removal after kidney transplantation may reduce length of stay. Foley catheters may cause patient discomfort and inhibit early ambulation.^7^ Early ambulation mitigates risks of deep venous thrombosis, atelectasis, and pneumonia. Length of stay is the most significant contributor to the cost of transplantation.^8^ Early catheter removal might be an inexpensive way to reduce length of stay and the overall cost of kidney transplantation while benefiting patient recovery and satisfaction.^9^

In prior comparative studies (Table 1), a few have shown that Foley removal on posttransplant days 2 or 3 has had comparable urinary leakage to longer Foley durations.^3^^,^^4^ Only one study investigated Foley removal 1 day after kidney transplantation^6^; however, the study sample was entirely living-donor kidney recipients, limiting generalizability.

**Table 1:** Summary of Comparative Studies of Foley Catheter Duration in Kidney Transplantation

Study	Foley Duration (n)	Ureteral Stent	UVA	Living Donor	DGF^a^	Urine Leak	Foley Reinsertion	LOS
Siskind et al (2013)^6^	(81) =1 d (39) >1 d	NA	FT	100% 100%	NA NA	7.4% 7.7%^ns^	NA NA	NA NA
Bezherano et al (2022)	(186) =1 d (327) >1 d	12% 28%	FT	8.6% 6.1%	49% 48%^ns^	1.1% 1.2%^ns^	12% 5.8%^s^	47% 58%^s^
Cole et al (2007)^3^	(66) =2 d (75) >2 d	100% 100%	NA	0% 51%	33% 16%^s^	0% 0%^ns^	1.5% 2.6%^ns^	3.2 d 5.0 d^s^
Glazer et al (2009)^4^	(30) 3 d (27) >3 d	0% 0%	FT+	0% 0%	10% 44%^ns^	0% 0%^ns^	NA NA	R^2^ 0.41^ns^
Hoy (1985)^11^	(100) =2-3 d (168) ≥3 d	NA NA	NA	68% 0%	NA NA	NA NA	NA NA	NA NA
Akbari et al (2017)^2^	(66) <5 d (43) ≥5 d	100% 100%	NA	100% 100%	NA NA	NA NA	NA NA	NA NA

Abbreviations: DGF, delayed graft function; FT, full thickness; FT+, full thickness with nipple valve; LOS, length of stay; NA, not available; ns, not significant; s, significant; UVA, ureterovesicular anastomosis.

a Defined as need for posttransplant dialysis within 2 weeks^3^ or any time post-transplantation^4^

**Table 2:** Donor and Recipient Characteristics and Kidney Transplant Outcomes by Foley Duration

	Characteristic, % or Mean ± SD	Foley Day 1 (n=186)	Foley >Day 1 (n=327)	P
Recipient	Age, y	54.6 ± 12.9	54.4 ± 12.9	0.86
	Race, Black	51 (27.4%)	106 (32.4%)	0.50
	Sex, F	74 (39.8%)	125 (38.2%)	0.79
	Body mass index >30 kg/m^2^	97 (52.2%)	189 (57.85%)	0.25
	Ureteral stent used^a^	22 (11.8%)	92 (28.1%)	<0.01^b^
	Diabetes	76 (40.9%)	140 (42.8%)	0.73
	Previous kidney transplant	22 (11.8%)	47 (14.4%)	0.49
	Calculated panel reactive antibody >0%^c^	44 (23.7%)	116 (35.5%)	<0.01^b^
	Pretransplant dialysis >3 y	55 (30.0%)	92 (28.1%)	0.80
	Cold ischemia time ≥30 h^d^	111 (65.3%)	221 (72.0%)	0.15
Donor	Age, y	41.0 ± 14.2	40.0 ± 14.0	0.43
	Live donor	16 (8.6%)	20 (6.1%)	0.37
	Race, Black	24 (12.9%)	40 (12.2%)	0.93
	Sex, F	80 (43.0%)	125 (38.2%)	0.33
	Donation after circulatory death^d^	73 (42.7%)	112 (36.5%)	0.21
	Kidney donor profile index	52.8 ± 22.0	52.0 ± 23.5	0.17
Outcomes	Urine leak	2 (1.1%)	4 (1.2%)	>0.99^e^
	Catheter reinsertion	22 (11.8%)	19 (5.8%)	0.02^b^
	Length of stay >4 d	88 (47.3%)	189 (57.8%)	0.02^b^
	Delayed graft function	91 (48.9%)	158 (48.3%)	0.96
	30-d readmission	47 (25.3%)	89 (27.2%)	0.70
	90-d bacteriuria^f^	90 (48.4%)	162 (49.5%)	0.87

Abbreviation: SD, standard deviation.

a Stent usage: was determined by surgeon preference and was generally employed universally or very minimally. The frequency of stent usage by surgeon was Surgeon A 4.6%, B 0%, C 100%, D 95%, E 100%, F 4.0%

b Significant (*P* < 0.05).

c Calculated panel reactive antibody: peak percent of antibodies against human leukocyte antigens identified before transplantation.

d Deceased donor only (n = 171 vs 307)

e Fisher exact test

f Bacteruria was defined as ≥10 white/cells/high-power field, with 0-3 squamous epithelial cells/high-power field, and a predominant organism at ≥10^3^ or mixed nonspecified organisms at ≥10^5^ organisms/mL or with ≥10^4^ yeast/mL in patients with functioning allografts without indwelling Foley or percutaneous nephrostomy. All patients received antibiotic prophylaxis for 3 months posttransplantation consisting of trimethoprim-sulfamethoxazole double strength 3 times a week or dapsone or atovaquone if sulfa allergic.

Over the past 5 years, our kidney transplant program has progressively encouraged early Foley removal through practice guidelines, as previously described.^9^ In this study, we examined differences in outcomes after Foley durations of 1 day (d1) versus longer (>d1) among recipients of predominantly deceased-donor kidneys, specifically focusing on urinary leak and length of stay. Institutional review board approval was obtained (University at Buffalo 00001969) and informed consent waived before commencing this retrospective study.

Single-center data of consecutive adult kidney-only transplant recipients between June 2014 and May 2020 were retrospectively reviewed with follow-up of 3 months minimum. Exclusions were death or graft failure within 30 days of kidney transplantation (none of whom had urine leaks), Foley not placed, and unknown Foley duration because of nonfunctional electronic medical records during an institutional cyberattack. Foley duration was determined by the operating surgeon, who in our program employs the full-thickness ureteroneocystostomy technique.^10^ Outcomes assessed were as follows: (1) urine leak (defined as wound creatinine 2× serum, renal scintigraphy scan showing leak, or nephrostomy, stent, or reoperation for urine leak), (2) Foley reinsertion or intermittent catheterization, (3) hospital length of stay greater than median, (4) 30-day readmission, and (5) 90-day bacteriuria. Comparisons were examined with Fisher exact or χ^2^ tests for categorical and unpaired *t* tests for continuous variables.

Foley d1 recipients (n=186) versus Foley >d1 recipients (n = 327) were significantly less likely to have pretransplant antibodies against human leukocyte antigens and have a ureteral stent placed at transplantation (Table 2).^2^, ^3^, ^4^^,^^6^ Urine leak occurred in 1.1% of Foley d1 and 1.2% of Foley >d1 recipients (*P* > 0.99). The d1 group was significantly more likely to experience Foley reinsertion or intermittent catheterization after removal (11% vs 5%, *P* = 0.02) and less likely to have length of stay >4 days (47% vs 57%, *P* = 0.03). There were no significant differences in 30-day hospital readmission and 90-day bacteriuria.

Our findings add to the literature examining shorter Foley duration after kidney transplantation.^3^^,^^4^^,^^6^ We found similar urine leak rates between groups with Foley durations of 1 day versus >1 day. Prior studies have also found similar urine leak occurrence with short Foley durations of 1-3 days versus longer durations, with or without the use of ureteral stents, and nonsignificant differences in downstream events of Foley reinsertion^3^ and urinary tract infection.^4^ Notably, a full-thickness ureteroneocystostomy was employed in all but one of these studies, potentially suggesting surgeon confidence in early Foley removal with this technique.

We found that shorter duration of urethral catheterization was associated with shorter length of hospital stay. Similarly, Cole et al^3^ noted significantly shorter length of stay with 2-day versus >2-day Foley durations. Glazer et al^4^ noted a trend toward lower length of stay with shorter (3-day) versus longer (>3-day) Foley duration. The lower length of stay in these studies may have been enabled by an absence of urine leak in both groups and did not appear to be influenced by differential rates of delayed graft function between groups.

The generalizability of our findings may be limited by type of ureterovesicular anastomosis, which may differ among centers and surgeons. Multivariate analyses were not performed; potential associations can only be inferred. Use of ureteral stent and timing of Foley catheter removal and rationale for reinsertion was subjective and not protocol-based.

In conclusion, kidney transplant recipients who underwent Foley catheter removal on postoperative day 1 did not experience higher leak rates than those with longer Foley durations in this single-center study with low ureteral stent usage and the use of the full-thickness ureteroneocystostomy technique. Despite greater Foley reinsertion for urinary retention, the Foley removal day 1 group had reduced length of stay and similar 30-day readmission and 90-day bacteriuria.
